# Global Burden of Human Metapneumovirus: Bridging Gaps in Prevention, Diagnostics and Treatment

**DOI:** 10.1002/puh2.70094

**Published:** 2025-08-01

**Authors:** Victor Abiola Adepoju, Qorinah Estiningtyas Sakilah Adnani, Safayet Jamil, Masoud Mohammadnezhad, Abdulrakib Abdulrahim

**Affiliations:** ^1^ Department of HIV and Infectious Diseases Jhpiego, An Affiliate of John Hopkins University Abuja Nigeria; ^2^ Department of Public Health Faculty of Medicine, Universitas Padjadjaran Bandung West Java Indonesia; ^3^ Department of Public and Community Health Faculty of Medicine and Health Sciences, Frontier University Garowe Puntland Somalia; ^4^ Department of Public Health Daffodil International University Dhaka Bangladesh; ^5^ Faculty of Health, Education and Life Sciences Birmingham City University Birmingham United Kingdom; ^6^ Department of Medical Microbiology Faculty of Medicine and Health Sciences Universiti Putra Malaysia Serdang Selangor Malaysia

**Keywords:** global health | human metapneumovirus (hMPV) | respiratory virus | viral pneumonia

## Abstract

Human metapneumovirus (hMPV) is a respiratory virus with a significant impact, particularly on the paediatric population, yet it remains an under‐recognized contributor to respiratory infections globally. Recent epidemics in China, India and Europe underscore its clinical burden, with severe cases linked to bronchiolitis, pneumonia and exacerbations of chronic conditions. Despite its impact, hMPV remains underdiagnosed due to limited access to molecular diagnostics and routine surveillance, particularly in low‐ and middle‐income countries. The absence of antivirals or vaccines exacerbates its public health challenge. Advances in multiplex diagnostics and vaccine development offer hope but require sustained global investment. Addressing gaps in prevention, diagnostics and treatment is critical to mitigating hMPV's growing threat and ensuring equitable healthcare outcomes.

## 1 Introduction

Human metapneumovirus (hMPV) is a respiratory virus first discovered in the Netherlands in 2001 [1]. Since its identification, it has often been overshadowed by more well‐known respiratory pathogens such as respiratory syncytial virus (RSV) and influenza virus. However, in recent months, the global health community has observed a growing concern regarding its public health impact. Reports of increased hMPV cases from China, India and Europe during the winter season highlight its underestimated role in respiratory infections [2, 3]. Similar patterns have also been documented in the United States, where national surveillance shows a 17% rise in hMPV‐related paediatric hospitalizations during January–March 2025 compared with the same quarter in 2023 [4]. This suggests a broader global trend, although it remains unclear whether the observed increase reflects improved detection, seasonal variation or a genuine surge in incidence. This rising trend, coupled with its clinical severity, calls for a paradigm shift in how we understand, monitor, manage and prevent its spread.

## 2 Epidemiology: A Global Burden on the Rise

hMPV is a single‐stranded RNA virus in the Pneumoviridae family, closely related to RSV. It demonstrates a consistent seasonal pattern and peaks in winter and spring in temperate regions while circulating year‐round in tropical climates [5]. Individuals under the age of five are the most affected; however, cases have been reported across all age groups [6]. Globally, in 2018, hMPV was estimated to be associated with approximately 14 million cases of acute lower respiratory infections, over 640,000 hospitalizations and 7700 in‐hospital deaths among children under five [6]. Serological evidence indicates that nearly every child contracts hMPV by age five [1, 7, 8, 9]. However, in adults above 64 years, hMPV was estimated to cause approximately 470,000 hospitalizations globally in 2019, with more than half of the cases reported in low‐ and middle‐income countries (LMICs) [10]. Recent epidemics have revealed its significant impact: hMPV accounted for 6.2% of respiratory illness tests and 5.4% hospital admissions in China during the late 2024, which surpasses rhinovirus and adenovirus [2, 11]. Epidemiological data from the United States and China indicate a 17% increase in hMPV‐related paediatric hospitalizations in early 2025, respectively, as compared to the same period 2 years earlier, with similar patterns among elderly and immunosuppressed patients [4]. However, long‐term, denominator‐adjusted incidence curves are scarce. A recent CDC analysis covering July 2014–June 2024 concluded that continuous national time‐series adequate for year‐on‐year rate comparisons are still lacking for hMPV in most regions [12]. Consequently, current comparisons rely on 2‐ to 3‐year snapshots, limiting our ability to distinguish persistent upward trajectories from episodic spikes. This increase highlights hMPV as a growing public health concern, particularly for high‐risk groups. Similarly, scattered cases in India prompted public health advisories, resulting in approximately 90 reported cases in 2025 as of March 2025 [3, 13]. These data emphasize that hMPV can no longer be regarded as a benign infection but a critical contributor to the respiratory disease burden.

## 3 Clinical Manifestations: Beyond Mild Respiratory Symptoms

Although hMPV often presents as a common cold, its clinical spectrum extends to severe complications, particularly in vulnerable populations such as infants, the elderly and immunocompromised individuals [14]. Severe cases can lead to bronchiolitis, pneumonia and exacerbations of asthma or chronic obstructive pulmonary disease [15]. Alarmingly, co‐infections are rarely benign and can significantly exacerbate disease severity. For instance, Semple and colleagues long ago identified that co‐infection with both hMPV and hRSV is associated with severe bronchiolitis [16]. A study conducted in Liverpool, UK, reported high rates of hMPV‐RSV co‐infections in paediatric patients, highlighting the complex interplay between these viruses [17]. Similarly, co‐infection with hMPV and RSV has been shown to increase the risk of intensive care unit (ICU) admission, particularly in children under the age of five when compared to mono‐infection [18]. Such findings challenge the longstanding assumption that hMPV infections are predominantly mild [19] and emphasize its potential to strain healthcare systems, especially during peak respiratory virus seasons.

## 4 Gaps in Prevention, Diagnosis and Treatment of hMPV

One of the primary barriers to addressing hMPV is the lack of routine diagnostic testing. Molecular methods like RT‐PCR have significantly advanced our understanding of hMPV epidemiology, revealing its widespread circulation and genetic diversity [2]. Yet, these tools remain inaccessible in many LMICs, where respiratory infections already account for a significant portion of child mortality [5]. The absence of widespread testing perpetuates its underestimation in clinical settings and leads to missed opportunities for targeted interventions and contributes to unnecessary antibiotic use, which fuels antimicrobial resistance [3].

Unlike RSV and influenza, hMPV currently lacks approved antivirals or vaccines, leaving symptomatic management as the sole treatment option. This therapeutic void disproportionately impacts resource‐limited settings, where severe cases often result in preventable morbidity and mortality. Recent advances in vaccine development offer a glimmer of hope. For instance, the University of Oxford recently launched a Phase 1 trial for a dual RSV‐hMPV mRNA vaccine in infants [20]. However, the path from promising candidates to widespread implementation is fraught with challenges, including antigenic variability, logistical barriers and the need for sustained global investment. Gaps in hMPV prevention, diagnostics and treatment are elaborately presented in Table 1.

**TABLE 1:** Addressing gaps in human metapneumovirus (hMPV) prevention, diagnostics and treatment.

S/N	Domain	Current challenges	Proposed solutions
1	Limited surveillance/Underestimation	Underestimation due limited routine inclusion of hMPV in global respiratory surveillance programs especially in LMICs [3, 10]	Establish regional sentinel surveillance systems with basic laboratory capacity such as multiplex diagnostic platforms for hMPV testing, platforms alongside RSV and influenza to monitor co‐circulation and identify trends, starting with LMICs and high‐burden areas and gradually expand through partnerships with existing respiratory disease monitoring programs
2	Diagnostics	Low accessibility to RT‐PCR and antigen detection tools, particularly in LMICs [21]	Scale up affordable, rapid molecular diagnostics and explore partnerships with global health initiatives for equitable distribution
3	Treatment	Absence of antiviral therapies for hMPV, leading to reliance on symptomatic care [2, 6]	Accelerate clinical trials for potential antivirals and explore repurposing of existing drugs with similar mechanisms of action
4	Vaccination	No approved vaccines, with early candidates still in clinical trials [6]	Support sustained investment in vaccine research and ensure equitable access through collaborations with Gavi and other vaccine financing mechanisms
5	Public awareness	Low recognition of hMPV as a significant respiratory pathogen compared to RSV and influenza. Public awareness of hMPV remains significantly low and may cause individuals to delay seeking healthcare or neglect preventive measures [22, 23]	Launch global awareness campaigns to educate healthcare providers and the public on the clinical burden and risks of hMPV
6	Health system strain	Increased healthcare utilization during seasonal outbreaks due to severe cases and co‐infections with RSV or influenza [17]	Strengthen health systems with surge capacity planning, especially during peak respiratory seasons, and prioritize vulnerable populations for preventive strategies
7	Equity in access	Disparities in resource allocation for diagnostics and care, particularly in LMICs	Advocate for policy‐level changes to integrate hMPV testing and interventions into existing child and elderly health programs in resource‐limited settings

Abbreviations: LMIC, low‐ and middle‐income countries; RSV, respiratory syncytial virus.

## 5 The Way Forward

Effective response to the threat of hMPV necessitates its inclusion in broader respiratory disease strategies. Multiplex diagnostics, successfully scaled during the COVID‐19 pandemic, provide a blueprint for enhancing hMPV detection and differentiation from co‐circulating pathogens. Surveillance systems must incorporate hMPV with RSV and influenza to provide a comprehensive picture of respiratory disease dynamics. Equally important is the equitable development and deployment of vaccines. Lessons from RSV and influenza underscore the need for robust surveillance, genetic monitoring and multisectoral collaboration to overcome barriers in vaccine access. Public health campaigns should prioritize awareness of hMPV, particularly its risks to vulnerable populations, whereas policymakers must allocate resources to accelerate vaccine research and development.

## 6 Conclusion

hMPV is a significant respiratory pathogen that has been circulating in the human population for decades. However, the recent rise in reported cases particularly in China, India and Europe may be attributed to either an actual increase in prevalence or enhanced testing efforts, possibly compounded by declining population immunity. This situation underscores the urgent need for sustained global attention and research. Addressing this challenge requires a coordinated approach that bridges existing gaps in diagnostics, treatment and prevention. Policymakers, researchers and healthcare providers must take decisive action to mitigate the impact of hMPV through long‐term global surveillance and investment in targeted public health strategies.

## Author Contributions

**Victor Abiola Adepoju**: conceptualization, investigation, writing – original draft, visualization, methodology, writing – review and editing, project administration, supervision, resources. **Qorinah Estiningtyas Sakilah Adnani**: conceptualization, investigation, writing – original draft, writing – review and editing, resources, project administration, supervision. **Safayet Jamil**: conceptualization, writing – original draft, writing – review and editing, supervision. **Masoud Mohammadnezhad**: writing – original draft, writing – review and editing, resources. **Abdulrakib Abdulrahim**: writing – review and editing, resources.

## Conflicts of Interest

The authors declare no conflicts of interest.

## Data Availability

As this is not a research article, we did not use any primary data in this study. Therefore, data availability is not applicable.
